# Evaluation of the Starch Quantification Methods of* Musa paradisiaca, Manihot esculenta,* and* Dioscorea trífida* Using Factorial Experiments

**DOI:** 10.1155/2018/5901930

**Published:** 2018-11-13

**Authors:** J. J. Lafont-Mendoza, C. A. Severiche-Sierra, J. Jaimes-Morales

**Affiliations:** ^1^Universidad de Cordoba, Monteria, Colombia; ^2^Universidad de Cartagena, Cartagena de Indias, Colombia; ^3^Corporacion Universitaria Minuto de Dios (UNIMINUTO), Barranquilla, Colombia

## Abstract

**Background:**

Starch and its products are used in a variety of ways for both the food and nonfood industries. A factorial experiment is carried out with two factors to explain the behavior of the percentage of starch, where the factors correspond to the extraction method and to the raw material.

**Method:**

Three methods were used in triplicate: the first followed the official technique of the Association of Official Analytical Chemists (AOAC), to perform acid hydrolysis and quantification of starch by Titulation; the second method involved the colorful reaction with iodine using the UV equipment to measure the absorbance and calculate the percentage of starch; as a third method the FTIR was used, through which the concentration of the starch was calculated by the area under the curve obtained from the spectrum.

**Results:**

there is an effect of both the method and the raw material on the percentage of starch, while there was no effect of the interaction; the Tukey test indicates that the highest average percentage of extraction occurs with the extraction method by Titulation and with the starch of* Manihot esculenta*.

**Conclusion:**

It is used as raw material. The method of quantification of starch by UV-VIS spectroscopy was the best for the study samples because it presented less deviation in relation to the FTIR and Titulation methods.

## 1. Introduction

The starch is a food reserve polysaccharide predominant in plants, it is the most important and abundant from the commercial point of view, and it is the most common way to include carbohydrates in our diet; the foods rich in it are a good source of energy [1]; starch has been a fundamental part of the diet of man for many years; in addition to this, it has been given a large number of industrial uses so it is considered, after cellulose, the most important polysaccharide from the commercial point of view. This carbohydrate is found in various sources such as cereals, tubers, and some fruits, and although its composition does not change the properties, if it does so, this depends on the source from which it is extracted [2–4].

The starch comes from different sources with different crystalline structures; cereal grains such as corn, wheat, or rice are sources of starch, such as roots and tubers; for example, tapioca, cassava root, and potatoes are frequently used in the preparation of gluten-free foods; the reversible transformations between the starch and glucose that intervene in the maturation and after the harvest have a remarkable influence on the quality, and the concentration of the starch varies according to the state of maturity [5, 6]. Starch is also derived from legumes such as soybeans and chickpeas; starch granules form different grains that differ in size, ranging from 2 to 150 microns, and in shape, which can be round or polygonal [7].

Starch and its products are used in a variety of ways in both the food and nonfood industries. In food, it is used as an ingredient in different preparations and in the nonfood industry as a raw material for the elaboration of a wide range of products. The consumption of starch is destined approximately 75 percent to the industrial sector and 25 percent to the food sector [8–10].

In this work three raw materials are studied for the quantification of their starch. Among these is* Musa paradisiaca*, which belongs to the Musaceae family, grows abundantly in many developing countries, and as food is considered one of the most important sources of energy for people who live in the humble regions of many countries [11]. Next, the* Manihot esculenta *is detailed, it is a perennial woody plant; its stem is cylindrical and formed by knots (point where the leaf joins the stem) and internodes (portion of the stem between two knots). It can be multiplied better in a vegetative way; therefore the stems are important because when they are mature they are cut into stakes of 7 to 30 centimeters in length with which the plant propagates [12]. Finally we have* Dioscorea trífida*; in this the leaves are heart shaped, alternate or opposite, long stalked; their stems are winged or oval cross section with small flowers in clusters or in panicles of three sepals and three stamens; although they are food species, they are characterized by poor flowering [13].

Based on these three agricultural raw materials outlined above, it is necessary to quantify their starch by the three analytical methods most used in these cases: initially the Titulation (acid hydrolysis) typical of traditional analytical chemistry; UV spectroscopic methods; IR studied by instrumental analysis. To compare the methods of quantification, the support of statistical tools is necessary; however, a descriptive study is not sufficient for this purpose, due to the inherent variations in said process; therefore, the support of an inferential tool is necessary to identify the importance of the effect that these factors have on % starch. The experimental design is a very useful tool in the comparison of processes [14], while the factorial experiments allow you to observe the effect of several factors on a variable response, as well as the interaction of them [15].

Taking into account the fact that it is very important to know the properties of starch and determine how these vary depending on the source from which it is obtained, by means of this work we quantify* Paradisiaca Musa*,* Manihot esculenta*, and* Dioscorea trífida*, by means of Titulation, UV-VIS spectroscopy, and FTIR in order to demonstrate which of these raw materials provides the highest percentage of starch and which of the three techniques is most appropriate for each one.

## 2. Materials and Methods

### 2.1. Starch Removal

The method of wet starch extraction consisted basically of grinding the pulp and removing in liquid medium those components that are relatively larger, such as fibers and proteins, using sieves; subsequently, the elimination of the water by decantation is facilitated and the sedimented material was washed to eliminate the last different fractions of the starch and finally it was subjected to drying at room temperature. For the extraction of the starch, 2000 grams of each raw material was used, which was acquired from the market of the city of Monteria (Cordoba, Colombia). The method developed consisted of the following stages [16].

Washing: It was carried out using potable water.

Peeling: Separation of the pulp shell manually with knife. In the case of* Musa paradisiaca*, after peeling, it was disinfected with a solution prepared with 1% sodium hypochlorite for 10 minutes.

Maceration: The samples were divided into slices, and water was added to then decrease size to the maximum in a blender for two minutes for the case of* Musa paradisiaca *and* Dioscorea trífida*; for* Manihot esculenta *this had a grating process.

Sieving: The product that was obtained is a mixture of starch, water, proteins, minerals, and impurities. To separate them, this slurry was passed through sieves.

Decantation: The suspension obtained was deposited in a plastic container and left for 4 hours, then the supernatant was removed to ensure that all impurities had been removed, and the wet starch was washed and sieved three more times.

Drying: The wet starch was dried in the environment for a day and then steamed to refine it.

### 2.2. Quantification of Starch

The quantification of the starch content of the three raw materials studied was carried out using the Titulation methods (acid hydrolysis), spectroscopic analysis (UV-VIS), and IR with Fourier transform (FTIR), which are described below.

Titulation (acid hydrolysis): The technique 920.44 of the (AOAC, 1995) [17] was used for the acid hydrolysis of the starch; for this determination 5 g of macerated sample was used; the method of acid hydrolysis was tested, by means of which 20 mL of concentrated hydrochloric acid and 200 mL of distilled water were added to the sample of starch and heated for 2.5 h in a ball fitted with a condenser to avoid evaporation. It was then cooled and neutralized with sodium hydroxide in beads, transferred to a 250 mL flask, made up to volume with distilled water, and filtered. To 25 mL of the liquid obtained were added equal volumes of alkaline tartrate, copper sulfate prepared according to method 923.09 (AOAC, 2000) [18], and distilled water, giving a final volume of 100 mL. This solution was carefully heated for 4 minutes, followed by 2 minutes of boiling; when hot, it was filtered under vacuum; after filtering the entire sample, it was washed with water at 60°C and with ethyl alcohol, to accelerate the subsequent drying process. It was dried in an oven at 100°C until constant weight; then the filter paper was weighed obtaining the content of copper oxide (I) and, based on it, the grams of glucose and subsequently the percentage of starch; the procedure was repeated by triplicate.

Spectrophotometric analysis (UV-VIS): UV-Vis spectroscopy was one of the first physical methods applied to quantitative analysis and the determination of molecular structures. The technique of UV- Vis spectroscopy is widely used in quantitative analysis, although in qualitative analysis, in the determination of structures, it is surpassed by other techniques, such as infrared spectroscopy and nuclear magnetic resonance. The absorption of UV-Vis radiation by molecules and compounds is due to the electronic transitions of certain groups that make them called chromophobes. These are unsaturated groups, with a large number of electrons responsible for the absorption of UV and Vis radiation. This absorption of energy causes electronic transitions and the usual formation of excited states. Based on the type of electronic transitions that occur, the absorbing species can be classified and, in addition, it is possible to correlate the spectral behavior of a certain species with its chemical characteristics. The electronic transitions that originate from the absorption of UV-Vis radiation can be divided into electronic transitions between orbitals, charge transfer transitions, and ligand field transitions. This procedure of colorful reaction with iodine can only be used when the starch is completely dissolved (gelatinized). For this procedure, 3 mL of the starch solution was placed in a test tube and 1 mL of an I / KI solution was added; then the intensity of the blue color produced in a 600 nm spectrophotometer was measured against a reagent blank.; the amount of starch present in the sample was calculated from a standard curve prepared in the range of 10-50 mg of soluble starch / mL, treated in the same manner as the test sample. For each sample of* Manihot esculenta *starch,* Musa paradisiaca*, and* Dioscorea trífida*, the procedure was repeated in triplicate [19].

IR spectroscopic analysis with Fourier transformed (FTIR): The basis of near infrared spectroscopy consists essentially in the emission of a monochromatic beam of light on the sample, which, depending on its composition and the nature of the bonds present in its molecules, will perform a selective absorption of energy and it will reflect another determined quantity, which is quantified by some detectors present in the NIR instrument and will be used to indirectly quantify the amount of infrared energy absorbed. In this way, the spectra collected in the infrared region are represented graphically as the energy absorbed as a function of the wavelength. In this regard it should be noted that only molecules or part of molecules that vibrate with a frequency similar to that of the incident energy will absorb infrared radiation, so that their vibrational and rotational states are modified, with vibrations of light atoms taking place with strong molecular links. These characteristics correspond to the functional groups C-H, O-H, and N-H of the organic compounds that are part of both plant and animal tissues. For this reason, NIR spectroscopy is practically oriented to the determination and quantification of organic compounds that present the functional groups described above; consequently, from the spectra obtained in the infrared region, information about the chemical composition of the sample analyzed can be obtained. Therefore, the relationship of the energy absorbed with the analytical composition or known characteristics of the calibration samples allows obtaining prediction models for the automatic and instantaneous analysis of thousands of samples. In this sense it can be said that in the last ten years some analytical services based on NIRS technology have been consolidated within the agri-food industry, and many others have been created, both public and private, since NIRS technology or spectroscopy in near infrared is an analytical technique widely implemented in agri-food industries as a support tool for the quality control of raw materials and products, mainly due to its rapid analysis, low cost per sample, simplicity of handling, similar precision to the reference method, no or little preparation of the sample for analysis, etc. For the quantification of starch we proceeded to make a calibration curve and analyzed the spectra of* Musa paradisiaca *starch,* Manihot esculenta*, and* Dioscorea trífida *samples, using an infrared spectrophotometer with transformed Fourier equipped with a KBr tablet system and total reflectance system attenuated at a temperature of 25 ± 2°C; the solid sample of starch will be introduced in KBr tablets with different concentrations of starch, and finally it will be measured in the equipment in the region of 400 to 4,000 cm-1. We calculate the problem concentration from a calibration curve prepared in the range of 20-100 mg of starch/mg of KBr. For each sample of* Manihot esculenta *starch,* Musa paradisiaca*, and* Dioscorea trífida*, the procedure was repeated in triplicate [20].

### 2.3. Statistical Analysis

Initially, the descriptive statistics of the percentage of starch are made, with respect to the raw material used and the extraction method; subsequently, an analysis of variance is carried out using a factorial experiment 3^∧^2 with three repetitions, to determine the effect of the extraction method, the raw material, and the interaction between raw material and extraction. Finally, the Tukey test and the media graphs are performed to observe the behavior of the factors, a strategy of data analysis similar to that carried out by Baldiris et al. (2017) [21].

## 3. Results and Discussion

Next, we present the analysis of the results obtained by the procedures used to quantify the starch content of the three raw materials studied (*Musa paradisiaca, Manihot esculenta, *and* Dioscorea trífida*) which were carried out using the Titulation methods (acid hydrolysis), analysis spectroscopic (UV- VIS), and IR with Fourier transform (FTIR).

### 3.1. Descriptive Statistics

According to the results obtained in Table 1, we proceed to calculate descriptive statistics as average, standard deviation, and coefficient of variation (CV).

The results of Table 2 show that the Titulation method is the one that provides the highest averages of percentage of starch in the different raw materials, while the percentages of average starch extracted from the* Manihot esculenta* are those that show the highest values. With respect to the variations, low dispersions are observed in the different methods and raw materials (CV <12%); however, the greatest variations are observed in the method by Titulation and with the raw material of the* Dioscorea trífida*.

### 3.2. Analysis of Variance

**Figure 1:**
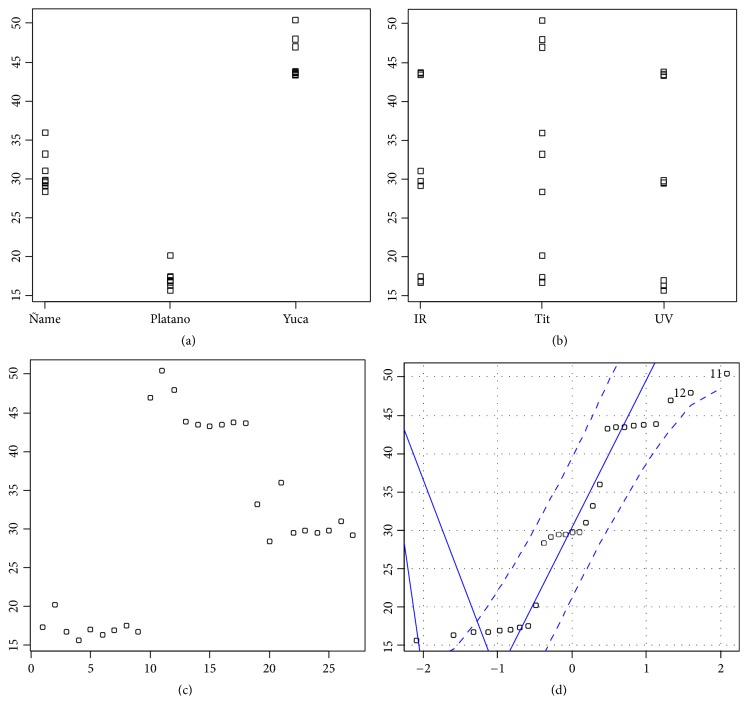
Diagnostic graphs of the assumptions of the variance analysis model.

Before performing experimental design for starch percentages, the diagnostic graphs of the assumptions of the model are made as illustrated in Figure 1.

Figures 1(a) and 1(b) correspond to the dispersion graph by raw material and by method, respectively; in them it is observed that the behavior of the variations of the groups is very similar, which corroborates the observed deviations obtained in the descriptive measures. With respect to the independence of the data, the chronological sequence graph, Figure 1(c) shows subgroups between the data, which can be explained by a possible effect of the factors considered in the study, and finally Figure 1(d) shows the normal probability graph, where it is observed that the data conform to this behavior. The above indicates that the analysis of variance can be performed since the data meet the specifications for it, as shown in Table 3.

Table 3 shows the analysis of variance for the percentage of starch, where a factorial experiment is carried out under a completely randomized design. The P-Values of the test indicate that there is an effect both of the extraction method and of the raw material used (P-Values less than 0.01); with respect to the interaction, it is observed that it was not significant, since the P-Value is 0.32. The Tukey test is then carried out, both for the method and for the raw material, to observe the behavior of the said differences.

**Figure 2:**
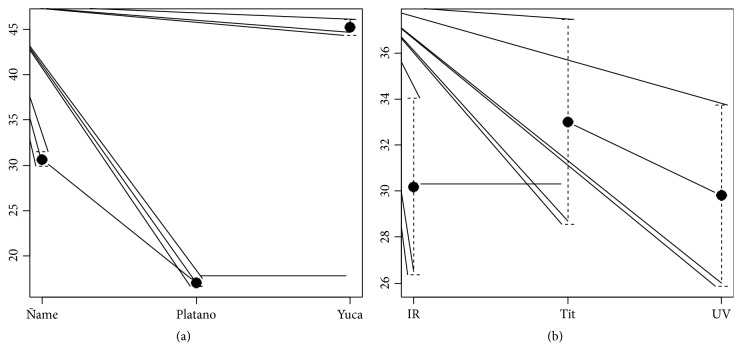
Average chart for the percentage of starch by raw material and by extraction method.

The means test for the different raw materials shown in Table 4 indicates that there is a highly significant difference with respect to the percentage of starch extracted; the average chart, Figure 2(a), shows that for* Manihot esculenta *there is a higher average extraction of starch percentage.

For the methods, there is a highly significant difference between the Titulation method and the UV and IR methods (P-Values <0.01) as noted in Table 5; the said method has the highest average value with respect to the percentage of starch; see Figure 2(b).

## 4. Conclusions

According to the review of the literature, the results shown, and their discussion, the following conclusions can be obtained:* Manihot esculenta *presented a higher yield of starch extraction, turning it into a potential source for this product as an alternative in the food industry with respect to the other raw materials studied which presented a lower extraction yield. The assumptions of normality, homogeneity of variance, and independence are fully met. As for the experimental design, it indicates that there is a highly significant effect of the raw material and the extraction method on the percentage of starch, but not on the interaction. Finally, the means tests show that the highest average starch percentages are obtained for the extraction method by Titulation and for the starch based on* Manihot esculenta.*

## Figures and Tables

**Table 1:** Starch percentages for the different raw materials using the different methods.

Raw material	Method		
	Titulation (%)	UV (%)	IR (%)
*Musa paradisiaca*	17,33	15,63	16,85
	20,15	16,97	17,47
	16,69	16,27	16,7

*Manihot esculenta*	46,95	43,82	43,44
	50,43	43,45	43,74
	47,95	43,28	43,62

*Dioscorea trífida*	33,16	29,52	29,76
	28,35	29,77	30,99
	35,94	29,48	29,15

**Table 2:** Descriptive statistics for the percentage of starch with respect to the method and the raw material.

Raw material	Statistics	Method		
		Titulation (%)	UV (%)	IR (%)
*Musa paradisiaca*	Average	18,06	16,29	17,01
	Deviation	1,84	0,67	0,41
	CV	10,20%	4,11%	2,40%

*Manihot esculenta*	Average	48,44	43,52	43,6
	Deviation	1,79	0,28	0,15
	CV	3,70%	0,63%	0,35%

*Dioscorea trífida*	Average	32,48	29,59	29,97
	Deviation	3,84	0,16	0,94
	CV	11,82%	0,53%	3,13%

**Table 3:** Analysis of variance for the percentage of starch depending on the method and the raw material.

Source of variation	Sum of squares	DF	F-Value	P-Value
Method	54.7	2	107.142	0.0008613*∗∗∗*
Raw material	3546.7	2	6.950.566	< 2.2e-16*∗∗∗*
Method: Raw material	12.6	4	12.394	0.3295978
Residuals	45.9	18		

*∗∗∗*: Highly significant difference (P-Value <0.01).

**Table 4:** Tukey test among raw materials.

	Difference	lwr	upr	P-Value
*Musa paradisiaca -Dioscorea trífida*	-1.356.222	-1.548.394	-1.164.050	0,0000*∗∗∗*
*Manihot esculenta- Dioscorea trífida*	1.450.667	1.258.494	1.642.839	0,0000*∗∗∗*
*Manihot esculenta- Musa paradisiaca*	2.806.889	2.614.717	2.999.061	0,0000*∗∗∗*

*∗∗∗*: Highly significant difference (P-Value <0.01).

**Table 5:** Tukey test among raw materials.

	Difference	lwr	upr	P-Value
Tit-IR	28.033.333	0.8816116	4.725.055	0.0042229*∗∗∗*
UV-IR	-0.3922222	-23.139.440	1.529.500	0.8621970
UV-Tit	-31.955.556	-51.172.773	-1.273.834	0.0013494*∗∗∗*

*∗∗∗*: Highly significant difference (P-Value <0.01).

## Data Availability

The data used to support the findings of this study are available from the corresponding author upon request.
